# A somatic hypermutation–based machine learning model stratifies individuals with Crohn's disease and controls

**DOI:** 10.1101/gr.276683.122

**Published:** 2023-01

**Authors:** Modi Safra, Lael Werner, Ayelet Peres, Pazit Polak, Naomi Salamon, Michael Schvimer, Batia Weiss, Iris Barshack, Dror S. Shouval, Gur Yaari

**Affiliations:** 1 The Alexander Kofkin Faculty of Engineering, Bar Ilan University, 5290002, Ramat Gan, Israel;; 2 Bar Ilan Institute of Nanotechnology and Advanced Materials, Bar Ilan University, 5290002, Ramat Gan, Israel;; 3 Institute of Gastroenterology, Nutrition and Liver Diseases, Schneider Children's Medical Center of Israel, Petah Tikva 4920235, Israel;; 4 Sackler Faculty of Medicine, Tel Aviv University, Tel Aviv 6997801, Israel;; 5 Pediatric Gastroenterology Unit, Edmond and Lily Safra Children's Hospital, Sheba Medical Center, Ramat Gan 5262100, Israel;; 6 Institute of Pathology, Sheba Medical Center, Ramat Gan 5262100, Israel

## Abstract

Crohn's disease (CD) is a chronic relapsing–remitting inflammatory disorder of the gastrointestinal tract that is characterized by altered innate and adaptive immune function. Although massively parallel sequencing studies of the T cell receptor repertoire identified oligoclonal expansion of unique clones, much less is known about the B cell receptor (BCR) repertoire in CD. Here, we present a novel BCR repertoire sequencing data set from ileal biopsies from pediatric patients with CD and controls, and identify CD-specific somatic hypermutation (SHM) patterns, revealed by a machine learning (ML) algorithm trained on BCR repertoire sequences. Moreover, ML classification of a different data set from blood samples of adults with CD versus controls identified that V gene usage, clusters, or mutation frequencies yielded excellent results in classifying the disease (F1 > 90%). In summary, we show that an ML algorithm enables the classification of CD based on unique BCR repertoire features with high accuracy.

Inflammatory bowel disease (IBD) is a group of chronic relapsing remitting disorders, typically manifesting as abdominal pain and diarrhea, that affect millions of people worldwide and require the administration of medications to control disease activity and prevent complications (Baumgart and Sandborn 2012; Ordás et al. 2012). There are two subtypes of IBD: Crohn's disease (CD), in which inflammation can involve any part of the gastrointestinal tract and is transmural, in contrast to ulcerative colitis, in which inflammation is confined to the colon and is considered superficial. Significant progress has been made in recent decades in our understanding of the processes that lead to IBD: These disorders develop in genetically susceptible hosts owing to a dysregulated immune response to microbial triggers or environmental factors (Chang 2020). The dramatic increase in IBD rates in the past couple of decades could be explained by changes to the “exposome,” including features that characterize the Western world, such as consumption of processed foods and high rates of antibiotic utilization (Kaplan 2015; Kaplan and Windsor 2021).

The immune response in IBD consists of multiple cell lineages from both innate and adaptive immune subsets. These include effector cells that augment inflammation (e.g., Th1, Th17 in CD) (Brand 2009) in contrast to anti-inflammatory populations, such as regulatory T cells that counteract the function of effector cells and suppress the hyperinflammatory response. A key feature of the adaptive immune system is a highly diverse immune repertoire of the T cell receptor (TCR), leading to billions of different clones that can bind unique antigens. The marked diversity in TCRs is formed following a complex rearrangement process of different gene segments, which involves recombination of variable (V), diversity (D), and joining (J) genes accompanied by deletion and insertion of random nucleotides, generating millions of unique TCRs (Nikolich-Žugich et al. 2004). Each TCR recognizes a specific antigen that can induce proliferation, cytokine secretion, and cell differentiation. Similarly, the B cell receptor (BCR) is formed after VDJ recombination. Somatic hypermutation (SHM) is an additional B cell–specific mode of diversification that relies on the introduction of mutations in BCR sequences. These mutations occur mostly at known “hotspots” in the DNA, which are concentrated in hypervariable regions (Pham et al. 2003). SHM is initiated by activation-induced deaminase (AID), which generates C-to-U mismatches on single-stranded DNA. A second arm involves Polymerase eta (Polη), a low-fidelity DNA repair enzyme that introduces mutations while elongating the broken DNA (Zeng et al. 2001).

High-throughput sequencing (HTS) platforms, developed in the past decade, enable a detailed assessment at the single-cell level of the TCR and BCR repertoires. We and others showed that individuals with IBD show oligoclonal T cell expansion with decreased diversity, mainly in the inflamed intestine but also in the blood to a lesser degree (Chapman et al. 2016; Doorenspleet et al. 2017; Günaltay et al. 2017; Werner et al. 2019). Studies focusing on the BCR repertoire in CD are sparse. Bashford-Rogers et al. (2019) compared BCR repertoires between several autoimmune diseases, and identified an increase in clonality in CD that was dominated by the IgA isotype, together with skewed use of the immunoglobulin heavy-chain V (*IGHV*) genes. Whether and how SHM is impaired in CD have not yet been explored.

Although CD is considered predominantly a T cell–mediated disease, individuals with CD also show an abnormal humoral response (Castro-Dopico et al. 2020). B cells are present in the ileum of healthy individuals, as well as in CD patients (Spencer and Sollid 2016; Martin et al. 2019). In CD patients, abnormal infiltration of B cells to mucosal sites of the intestine and atypical production of IgG and IgA (auto) antibodies to both bacterial and non-bacterial (auto)antigens have been observed (Chen et al. 2020). In-depth assessment of the BCR repertoires could provide more functional data on the role of B cells in mediating the hyperinflammatory response in CD (Pabst et al. 2015).

Current biomedical massively parallel sequencing experiments generate immense amounts of data, raising the need for new approaches to analyze and make data-driven recommendations, decisions, and predictions. Artificial intelligence (AI) and, specifically, machine learning (ML) approaches have been developed to integrate large amounts of data and generate signatures that could be used for classification of diseases or prediction of outcomes (Ostmeyer et al. 2017, 2019; Eliyahu et al. 2018; Greiff et al. 2020; Shemesh et al. 2021; Shoukat et al. 2021; Safra et al. 2022).

One example of a clinically relevant immense data set generated by a single experiment is sequencing of the BCR repertoire. Because of the longevity of immune memory, massively parallel sequencing of adaptive immune receptor repertoires (AIRR-seq) provides detailed insights into adaptive immune function (Laserson et al. 2014; Stern et al. 2014; Wendel et al. 2017; Pogorelyy et al. 2018; Puelma Touzel et al. 2020). However, AIRR-seq experiments sample only a small fraction of the huge diversity of the entire immune repertoire, and thus, estimations are made based on signals within these sampled repertoires. These are largely sampled from the most frequent BCR or TCR clusters and clones (Yaari and Kleinstein 2015). Many attempts involve dismantling the hypervariable regions, also termed complementarity determining regions (CDRs), to quantify features of the whole repertoire, for example, by breaking down the AA sequences of CDR3s into short stretches of *k* AAs (*k*-mer) and counting their frequencies (Ostmeyer et al. 2019; Ostrovsky-Berman et al. 2021; Shemesh et al. 2021).

In the current study, we performed an in-depth analysis of the BCR repertoire using HTS in the inflamed intestine of pediatric patients with CD compared with age-matched controls. In addition, we interrogated the BCR heavy-chain repertoires of blood samples from individuals with CD versus controls, as illustrated in Figure 1. We used ML tools to identify unique BCR-related signals that were used for the prediction of CD. The use of ML enables deciphering complex signals hidden in sequenced data.

**Figure 1.**
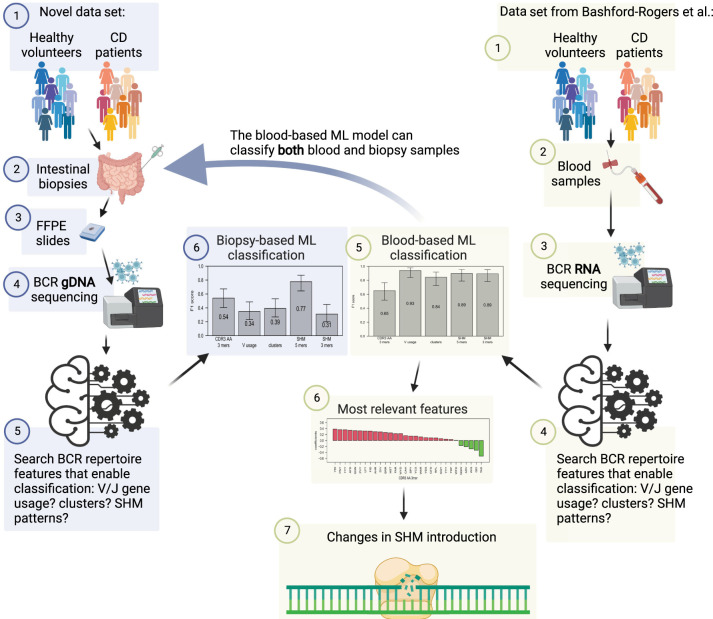
Study design. A new data set of BCR DNA sequencing from intestinal biopsies of 25 CD patients as well as 25 controls was created. ML algorithms were trained on many whole repertoire parameters to classify CD. Results were compared with the ability to predict CD on a previously published RNA blood cohort. Only changes in SHM patterns succeeded in CD classification on both data sets. This figure was created with BioRender (https://biorender.com).

## Results

### No statistically significant single feature differentiates between CD and control repertoires

**Figure 2.**
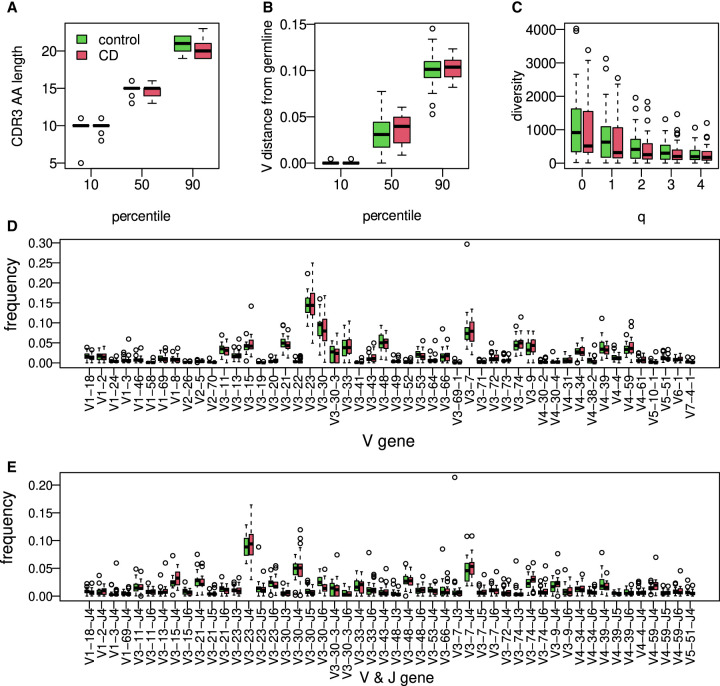
Characterization of ileal immune BCR repertoire features. (*A*) The 10, 50, and 90 percentiles of AA CDR3 length in individuals with CD compared with the controls. (*B*) The 10, 50, and 90 percentiles of V gene distances from germline in individuals with CD compared with the controls. (*C*) Boxplot showing calculated Hill diversity indexes upon different *q*-values between individuals with CD and the controls. (*D*) Boxplots showing V gene usage in individuals with CD and the controls; shown are the top 50 mean frequencies. (*E*) VJ gene usage comparison between individuals with CD and healthy ones; shown are the top 50 frequencies.

To characterize the BCR repertoire in the intestine of individuals with CD, we obtained formalin-fixed paraffin embedded (FFPE) biopsies from the ileum of 25 children with CD at the time of diagnosis, as well as samples from 25 aged-matched controls, pediatric patients referred to a colonoscopy for evaluation of abdominal pain or diarrhea. All controls showed a normal macroscopic and histologic evaluation of the gastrointestinal tract and did not have any past or present history of an immune-mediated disorder (IBD, celiac, diabetes, etc.). In contrast, all CD patients showed macroscopic inflammation of the terminal ileum with biopsies indicating chronic active ileitis (see Supplemental Fig. S1). There were no differences in the age of the patients at the time of colonoscopy (mean 13.8 ± 1.2 vs. 12.9 ± 1.4 yr) or in sex (13/25, 52% vs. 12/25, 48%) between the controls and CD patients, respectively. We generated BCR libraries for massively parallel sequencing from genomic DNA and performed a general exploratory data analysis (for technical details about the numbers of sequences and unique clones, see Supplemental Fig. S2). The study design is illustrated in Figure 1. Even though the CDR3 length distribution was slightly shorter in the CD group, the difference was not statistically significant (Fig. 2A), and neither were the differences in overall mutation frequency (Fig. 2B). We next inferred the clonal relationships between all BCR sequences in each individual, that is, sequences belonging to the same clone assumed to stem from a single ancestor. Diversity indices calculated from the inferred BCR clones of individuals with CD were slightly lower, although not significant (Fig. 2C), nor were the V or V–J gene usages (Fig. 2D,E). Collectively, we could not detect a single statistically significant feature that can be used to distinguish between the CD and control repertoires.

### Stratification of CD is possible based on SHM patterns in biopsy samples

**Figure 3.**
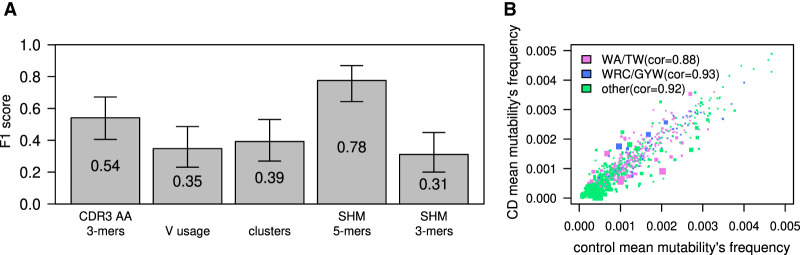
CD classification by tissue BCR sequencing. (*A*) F1 scores calculated for ML algorithm used to classify CD; estimations were made using a leave-one-out cross-validation method. The model was trained on frequency of 3-mers of CDR3's AA, V gene usage, clusters (same V and J genes and CDR3 length), or frequencies of a normalized DNA 5-mers/3-mers SHM model. Error bars show the confidential interval of 95% as calculated using binomial distribution. (*B*) Scatter plot showing mean frequency of each normalized 5-mer's mutability in controls and individuals with CD. The color of each dot represents whether it is part of one of two SHM hotspots (purple for the WA/TW hotspot, blue for the WRC/GYW hotspot, and green for the rest). The size of the dot is −Log(*P*-value) of differences between CD and controls. Spearman's correlation between mean frequencies of controls and individuals with CD for each group is shown.

To deepen our exploration of multifeature changes in the repertoires, we tested several ML algorithms to classify the two cohorts using a leave-one-out cross-validation scheme. We first applied an approach based on 3-mers of AAs from the CDR3, as well as an approach based on either V gene usage or the frequency of large BCR sequence clusters (same V gene, J gene, and CDR3 length; see Methods). These attempts resulted in low F1 scores of ∼0.5 (Fig. 3A, left three bars). Comparably, low F1 scores of about 0.57 were obtained using clusters with higher, 85%, identity in CDR3 AA sequences. We then explored whether SHM patterns can be used for such classification. We characterized the mutation patterns in each repertoire by building a 5-mer SHM model that describes the combined effects of SHM and affinity-dependent selection (see Methods). Targeting values were selected, and in each leave-one-out iteration, the top 30 differentiating features with the lowest *t*-test *P*-values in the training group were selected. Logistic regression was then trained on these data using LASSO and elastic-net regularization (Tibshirani et al. 2012; https://cran.r-project.org/web/packages/caret/index.html). Applying this method based on leave-one-out yielded ∼0.78 accuracy and F1 score (Fig. 3A). Note that classification is not based on reduced SHM frequencies, but on altered SHM patterns. As specific SHM patterns were found to be connected to specific V genes, we calculated SHM patterns to each of the five largest V families. The same algorithm as above, trained on partial data from sequences of specific V families, could not stratify individuals with CD from healthy individuals (Supplemental Fig. S4). Our SHM matrix was calculated by comparing BCR sequences to their inferred germlines. Calculating SHM compared with their ancestors from the inferred linage tree also did not succeed, and neither did consideration of the CDRs only (Supplemental Fig. S5).

In general, two highly mutated hotspot motifs are commonly observed in BCR sequences. One in the WRC/GYW (where W = {A, T}, Y = {C, T}, R = {G, A}, and the mutated position is underlined) consensus sequences is driven by the deaminase AID. The other is the WA/TW sites, driven mostly by the low-fidelity DNA Polη (Zeng et al. 2001; Yavuz et al. 2002). We noticed that most of the differences between individuals with CD and controls were in the WA/TW hotspots (Fig. 3B, pink dots), and therefore, we checked whether we can maintain the prediction accuracy based only on mutations in those hotspots. Training a logistic regression algorithm on WA/TW sites only resulted in lower F1 scores, but there was still a signal that enabled classification with partial success (F1 score of ∼0.65; see below). In contrast, considering only WRC/GYW sites completely abolished the signal that enables classification.

### ML model based on distinct blood BCR repertoire features enables CD stratification

**Figure 4.**
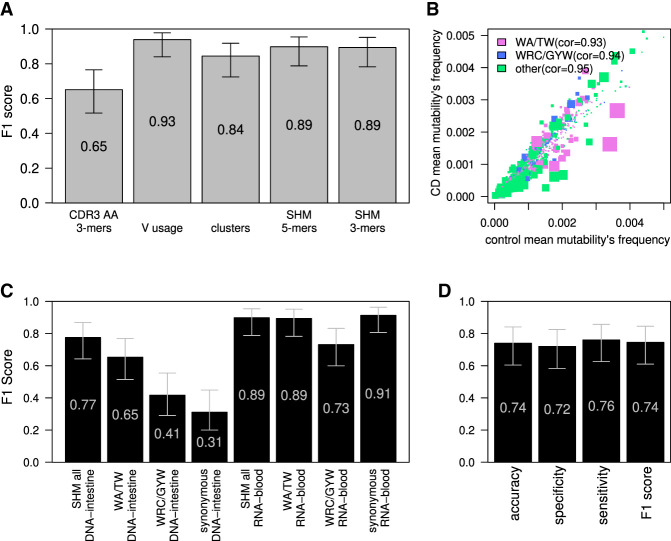
CD classification by blood BCR sequencing. (*A*) F1 score calculated for the ML algorithm used to classify CD; estimations were made using the leave-one-out cross-validation method. Algorithm was trained on frequencies of 3-mers of CDR3's AA, V gene usage, clusters (same V and J genes and CDR3 length), or frequencies of the normalized DNA 5-mers/3-mers SHM model. Error bars show the confidential interval of 95% as calculated using binomial distribution. (*B*) Scatter plot showing mean frequencies of each normalized 5-mers mutability in controls and individuals with CD. The color of each dot represents whether it is part of one of two SHM hotspots (purple for the WA/TW hotspot, blue for the WRC/GYW hotspot, and green for the rest). The size of the dot is −Log(*P*-value) of differences between CD and the controls. Spearman's correlation between mean frequencies of the controls and individuals with CD for each group is shown. (*C*) F1 score calculated in the same way as in *A*. Algorithm was trained on frequencies of all normalized DNA 5-mers SHM model, on only patterns within the WA/TW or WRC/GYW hotspots, or on all sites but using only synonymous mutations for SHM calculations. F1 score was calculated separately for the RNA-blood and the DNA-intestine cohorts. Error bars show the confidential interval of 95% as calculated using binomial distribution. (*D*) Barplot showing the accuracy, specificity, sensitivity, and F1 score of classification of the tissue cohort based on algorithm that was trained on the RNA-blood cohort. The algorithm used was logistic regression with LASSO and elastic net regularization (GLMNET), which was trained on substitutions from the RNA-blood cohort.

To validate our results, we applied ML to a high-quality BCR repertoire sequencing data set from Bashford-Rogers et al. (2019). The cohort comprises 29 adult controls and 24 adults with CD. These libraries were produced from peripheral blood samples, and RNA was used as a starting material instead of DNA in the intestine-derived libraries described above. We applied all ML algorithms used to classify the DNA-intestinal data set to this RNA-blood data set. We obtained better classification results compared with the DNA-intestine data set. ML classifications based on V gene usage, the presence of specific alleles in the genotypes (as inferred by the VDJbase pipeline) (see Supplemental Fig. S6; Gidoni et al. 2019; Omer et al. 2020), clusters, or mutation frequencies yielded excellent results (F1's of 0.94, 0.90, 0.84, and 0.90 respectively) (Fig. 4A). The only exception was classification based on AA 3-mers from CDR3, which resulted in a relatively low F1 score of ∼0.65 (Fig. 4A). Nevertheless, we obtained good results using either the whole SHM matrix and selecting for the top 200 features or using only the targeting model without external feature selection (F1 score of 0.9), suggesting again high signal levels. In an attempt to decipher the signal behind the success of classification based on mutability patterns, we applied the ML models to subsets of the mutability patterns. Here again, classification made based on mutation frequencies in WA/TW was much more accurate than that made based on WRC/GYW (Fig. 4B,C). This similarity between the two data sets encouraged us to train a logistic regression model on the RNA-blood samples and apply it to the DNA-intestine data set to test whether the model is transferable to different data types related to CD. This attempt resulted in an accuracy of ∼0.75 (Fig. 4D), suggesting some similarity in the CD-stratifying signal between the two data sets. The same algorithm was trained also on both data sets, with similar results, as shown in Supplemental Figure S7. The opposite direction, using a model built on the DNA-intestine data set, was unsuccessful in classifying the RNA-blood data set.

### Differences in SHM patterns between CD and control repertoires

**Figure 5.**
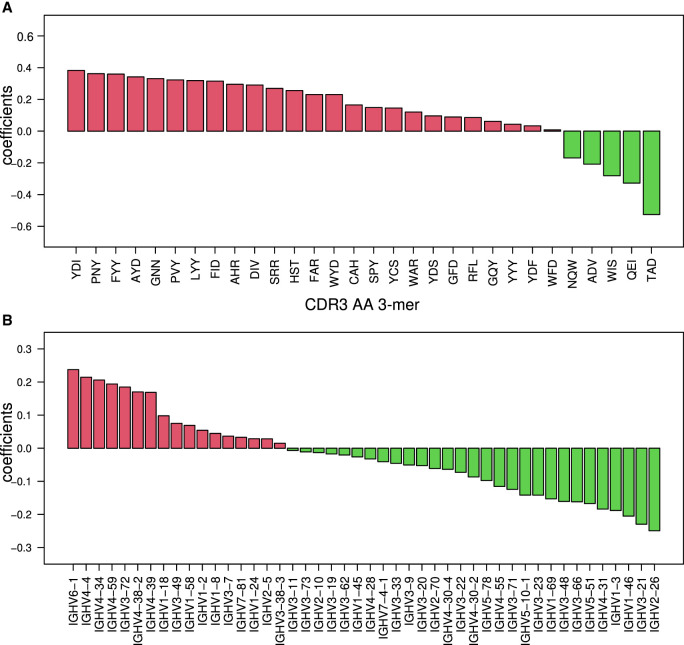
Top-ranked BCR repertoire–level features for RNA-blood CD stratification. (*A*) Bar graph showing the coefficients that were calculated on the RNA-blood data set with 3-mers of CDR3's AA. In red are coefficients in favor of CD; in green, coefficients against CD. (*B*) Bar graph showing the coefficients that were calculated on the whole RNA-blood data set with V gene usage. In red are coefficients in favor of CD; in green, coefficients against CD.

Classifications based on RNA-blood samples relied on clear differences in V-gene usage, SHM patterns, and CDR3 AA 3-mer frequencies between individuals with CD versus controls. The most important features for the classifications are shown in Figure 5, A and B. Specific D genes that are connected to specific CDR3 AA 3-mers are shown in Supplemental Figure S8. To investigate the differences in SHM patterns in individuals with CD versus controls, we simplified the model. Instead of using 5-mers, we used a 3-mer substitution matrix. We extracted 3-mer mutability data from the 5-mer tables and then trained the ML algorithm to find a CD-stratifying signal. We found the 3-mer-derived signal in the RNA-blood samples but not in the DNA-intestine samples (Figs. 3A, 4A). We also attempted to build an SHM model based only on synonymous mutations. Again, in the blood samples, we obtained successful classification but not in the intestine samples (Fig. 4C). Similarly, attempts to build the SHM model using a single representative from each clone succeeded in the RNA-blood data set but not in the DNA-intestinal cohort.

**Figure 6.**
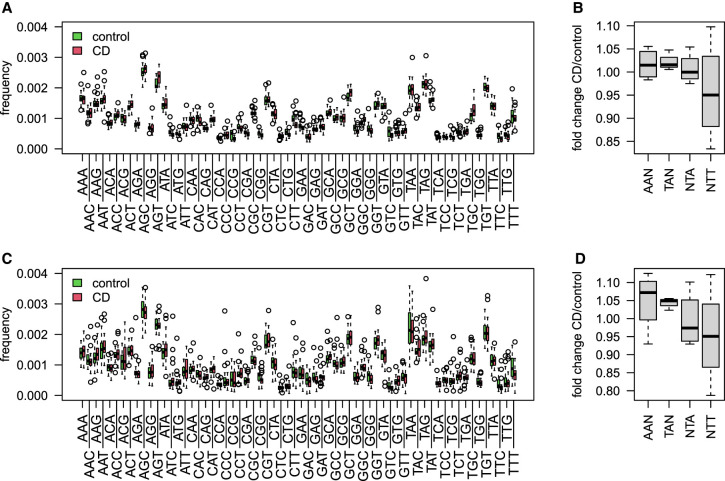
3-mer mutability model in CD patients compared with the controls. (*A*) Boxplots showing the frequency of all 3-mer mutability in the RNA-blood samples of individuals with CD compared with the controls. (*B*) Boxplots showing mutation frequency of individuals with CD normalized to the controls at WA/TW consensus sequences. (*C*) Boxplots showing the frequency of all 3-mers mutability in the DNA-intestine samples of individuals with CD compared with the controls. (*D*) Boxplots showing mutation frequency of individuals with CD normalized to the controls at WA/TW consensus sequences.

We further used the 3-mer matrix on both RNA-blood samples and the DNA-intestine samples to deeply assess the differences in CD substitution patterns at the WA/TW SHM hotspots. For this, we explored the differences in 3-mer mutability rates in all sites (Fig. 6A,C). Figure 6, B and D, shows boxplots of ratios between the mean mutability frequencies in CD and controls calculated for four generalized motifs (AAN/TAN/NTT/NTA). Both cohorts show a higher fold change in the two motifs AAN and TAN, whereas in the NTT motif, there is a decrease in mutability levels in CD patients. This observation points to a possible difference in the activity of Polη in CD or, more likely, a difference in the mutation patterns in the largest clusters represented in the CD patients compared with the healthy individuals (see Discussion).

## Discussion

In this study, we investigated BCR repertoire patterns in CD. We generated a novel BCR repertoire data set from FFPE ileal biopsies of children with CD and age- and sex-matched controls. Although no single repertoire-level feature differs significantly between CD and the controls, a multifeature ML model successfully stratified the groups (∼0.78 F1 score). We further explored recently published RNA-blood-derived BCR repertoire data and obtained even better stratification results (∼0.9 F1 score). Moreover, although classification using ileal biopsies succeeded only when using mutability patterns as features, in the RNA-blood BCR repertoire data, CD could be classified using numerous feature sets, including V gene usage, cluster frequencies, and AA 3-mers in the CDR3.

The differences in the ability to classify CD in these two data sets could stem from biological or technical factors. The biological differences between the experimental setups of the two studies are as follows: First is the libraries’ starting material. The blood libraries were generated from RNA, whereas the intestine data set was generated from DNA. In RNA libraries, B cells expressing high levels of antibodies are oversampled, whereas in DNA libraries, all B cells are sampled uniformly. Second is the tissue from which the samples were taken. In our data set, the samples were from ileal biopsies in contrast to blood. Third is the different age groups. The intestinal samples were obtained from children, and the RNA-blood samples came from individuals aged 20–77 yr. Fourth is the controls. The control group for the ileal CD samples was composed of pediatric patients referred to a colonoscopy for the evaluation of abdominal pain or diarrhea (because colonoscopies are not performed in healthy children), and the RNA-blood samples were taken from healthy individuals. Technical differences can be attributed to different library protocols: First, the RNA-blood data set includes unique molecular identifiers, which reduce PCR and sequencing errors (Shugay et al. 2014). This method currently cannot be applied to DNA. Second, the DNA-intestine data set was generated from FFPE tissues. Fragmented DNA in these preserved samples gives rise to higher levels of noise. The RNA-blood data set was generated using fresh samples. Third, we cannot rule out the possibility that batch effects in the RNA-blood samples between individuals with CD and controls give rise to the clear differences between the two groups. To reduce the effect of this potential artifact, we looked for a shared stratification signal across the two data sets. Even though the two data sets are different, as detailed above, when training the ML algorithm on the RNA-blood data set and applying it to the DNA-intestine data set, we still obtained an accuracy of about 0.75. This shared signal strengthens the conclusion that differences in SHM pattern are real biological signals that differ between individuals with CD and controls.

In the past few years, most attempts to classify diseases using AIRR-seq data were focused on the CDR regions. In this cohort, classification based on common clusters sharing the same V and J genes as well as 85% similarity in the sequences of CDR3 AA failed (accuracy of 0.53). However, classification based on SHM patterns did succeed. To our knowledge, this is the first time that an SHM-based model is used to classify clinical cohorts. Furthermore, the model reveals an underlying biological process that is altered in individuals with the disease. Two highly mutable SHM hotspots can be found in BCR sequences (Yaari et al. 2013). The first is in WRC/GYW consensus sites, which are attributed to the activity of the AID enzyme. The other is in WA/TW sites, which are attributed to mutations introduced by the low-fidelity DNA polymerase Polη. The classification results were significantly reduced when using only WRC/GYW substitution patterns but remained similar when using the WA/TW SHM patterns. Specifically, mutability frequencies in NTT sites of individuals with CD were slightly decreased compared with the controls. A similar trend is observed in both data sets. Notably, the overall SHM frequency is unaltered, but the mutational specificity is shifted such that mutations at the A/T motif are decreased, with a concomitant rise at the G/C motif. These results suggest either that the functionality of Polη in CD is altered or that WA/TW-containing patterns are more represented in the largest clusters of individuals with CD than in healthy individuals, likely because of selection during affinity maturation. Although the latter option seems more plausible, the successful classification of RNA-blood samples based on only silent mutations hints that affinity-dependent selection is not the whole story. Further studies are needed to identify the exact mechanism in CD, as well as to test whether such signal can predict a response to specific treatments. The revealed linkage between substitution patterns and CD may be causal or correlative. If the potential altered Polη activity is causal, then targeting Polη activity may have beneficial clinical implications. If, on the other hand, the observed changes are only correlative with the disease, they may still have a diagnostic and prognostic value. This is especially useful because the disease signal can be detected in the blood of patients, eliminating the need for an intestine biopsy to diagnose CD. Additional studies are required to better define these features in a large cohort of patients and to determine how they change in the course of the disease, during disease exacerbations, and during remission.

## Methods

### Collection of samples

The study was approved by the local Institutional Review Board at Sheba Medical Center (protocol 3312-16-SMC), and informed written consent was obtained appropriately. For the current study, we used FFPE ileal biopsies obtained for clinical purposes during colonoscopies of children <18 yr, archived in the Institute of Pathology at Sheba Medical Center. A description of the volunteers for the study can be found in Supplemental Table S1.

### DNA isolation from FFPE

DNA was isolated from FFPE samples based on a previously published protocol (Kresse et al. 2018), with some modifications. We used the same number of slides to prepare gDNA from each patient and used a relatively large number of slides to obtain a large pool of cells that will be representative of the population of B cells in the biopsy. To this end, 30 slices of 3.5 µm were obtained for each FFPE block. Paraffin was removed by addition of 165 µL ATL lysis buffer (Qiagen) and incubation for a few minutes at 90°C until a complete meltdown. This was followed by centrifugation at 19,000 rcf for 30 sec. After two cycles of Proteinase K digestion, an additional incubation for 1 h at 90°C was performed. Lastly, DNA isolation was performed using QIAamp DNA mini kit elution columns (Qiagen).

### IGH repertoire library generation and analysis

For each sample, 120 ng of genomic DNA was used in a multiplex, PCR-based assay kit (Lymphotrak). The kit contains different indices, each targeting conserved regions within the *IGHV* and the *IGHJ* regions. This enables a one-step PCR reaction and pooling of different samples. MiSeq libraries were prepared and sequenced using Illumina protocols. FASTA files were generated using the Lymphotrak kit attached software. The numbers of sequences in each cohort and correlation between number of sequences and number of clones are shown in Supplemental Fig. S2).

### Data processing

FASTA files were generated and then were aligned to *IMGT IGHV/D/J* genes (Brochet et al. 2008) using IgBlast (Ye et al. 2013). AIRR format output was generated using the MakeDb function from Change-O (Vander Heiden et al. 2014; Gupta et al. 2015). VJ gene usage and CDR3 AAs 3-mers, as well as CDR3 AAs length and V gene identity, were calculated using a custom-designed R script (R Core Team 2022). Diversity was calculated using the alphaDiversity function from the Alakazam R package (Gupta et al. 2015).

### Creating an SHM model

An SHM model was built using the function create TargetingModel from the shazam R package (Yaari et al. 2013), once for synonymous only mutations and once for nonsynonymous or synonymous mutations. For each repertoire, substitutions, mutability, and targeting values were collapsed into a single table. Tables from all repertoires were collapsed into a single table. Such a table can be found in Supplemental Table S2. The columns in these tables represent the probability of a mutation to occur in specific DNA 5-mers, as well as the probabilities of substitution to each of the other nucleotides. These motifs are shared between repertoires, and statistics can be made on values of the different groups.

### Training and estimation of ML algorithms

The leave-one-out cross-validation approach was used to estimate the F1 score, accuracy, sensitivity, and specificity of each model. LASSO models and elastic-net regularized generalized linear models (GLMNET) using the caret R package (Tibshirani et al. 2012; https://cran.r-project.org/web/packages/caret/index.html) were trained on tables containing repertoires data of V gene usage frequencies /V-J-CDR3 AA length large clusters (with frequency ≥ 0.001)/CDR3 AA 3-mer frequencies/SHM frequencies. Features were selected in each leave-one-out iteration according to the Student's *t*-test *P*-value. The top 30/200 features taken were determined for each algorithm run; all leave-one-out iterations used the same top feature selection. The GLMNET algorithm was then trained on the selected data, and a prediction was made for the leave-one-out test sample. F1 score, accuracy, sensitivity, and specificity were calculated after finishing all leave-one-out iterations and were calculated for the whole model. All results were compared with those obtained using shuffled labeled data, which gave the expected median of about 0.5 as shown in Supplemental Figure S3.

## Data access

The sequencing data generated in this study have been submitted to the NCBI BioProject database (https://www.ncbi.nlm.nih.gov/bioproject/) under accession number PRJNA788351. All code is available as Supplemental Code and at GitHub (https://github.com/saframodi/crohnData).

## Supplementary Material

Supplemental Material
