# Supplementary material for: A somatic hypermutation–based machine learning model stratifies individuals with Crohn's disease and controls
Source: Genome Res. 2023 Jan;33(1):71–9. doi: 10.1101/gr.276683.122 (PMC9977146; doi:10.1101/gr.276683.122)
Supplement: Supplemental Material [file supp_33_1_71__DC1.html]

A somatic hypermutation–based machine learning model stratifies individuals with Crohn's disease and controls — A somatic hypermutation–based machine learning model stratifies individuals with Crohn's disease and controls — Supplemental Material 

# A somatic hypermutation–based machine learning model stratifies individuals with Crohn's disease and controls

## Supplemental Material

- SupplementalCodes.zip
- Supplemental\_Table\_S1.xlsx
- Supplemental\_Table\_S2.xlsx
- Supplemental\_Material.pdf
