# Supplementary material for: A somatic hypermutation–based machine learning model stratifies individuals with Crohn's disease and controls
Source: Genome Res. 2023 Jan;33(1):71–9. doi: 10.1101/gr.276683.122 (PMC9977146; doi:10.1101/gr.276683.122)
Supplement: Supplemental Material [file supp_gr.276683.122_Supplemental_Material.pdf]

A somatic hypermutation-based machine learning model stratifies individuals with Crohn's disease and controls

Modi Safra[1,2,*] , Lael Werner[3,4,*], Ayelet Peres[1,2], Pazit Polak[1,2], Naomi Salamon[5], Michael Schvimer[6], Batia Weiss[4,5], Iris Barshack[4,6], Dror S. Shouval[3,4,†], and Gur Yaari[1,2,†]

[1]Faculty of Engineering, Bar Ilan University, 5290002, Ramat Gan, Israel
[2]Bar Ilan institute of nanotechnology and advanced materials, Bar Ilan University, 5290002, Ramat Gan, Israel
[3]Institute of Gastroenterology, Nutrition and Liver Diseases Schneider Children's Medical Center of Israel, Petah Tikva 4920235
[4]Sackler Faculty of Medicine, Tel Aviv University, Tel Aviv 6997801, Israel
[5]Pediatric Gastroenterology Unit, Edmond and Lily Safra Children's Hospital, Sheba Medical Center, Ramat Gan 5262100, Israel
[6]Institute of Pathology, Sheba Medical Center, Ramat Gan 5262100, Israel
[*]Joint first authors
[†]Joint last authors. Correspondence: dror.shouval@gmail.com, gur.yaari@biu.ac.il,

November 23, 2022

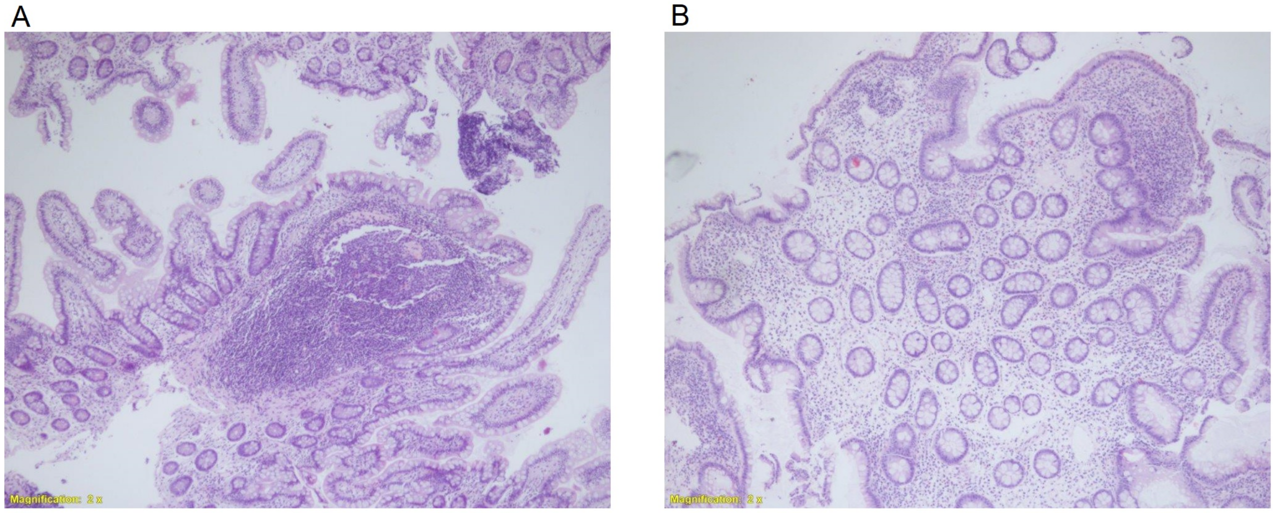

Figure S1: **Histologic examination of representative terminal ileum samples.** Hematoxylin and eosin staining of A. control, and B. inflamed (CD patient) terminal ileum samples. Original magnification X40.

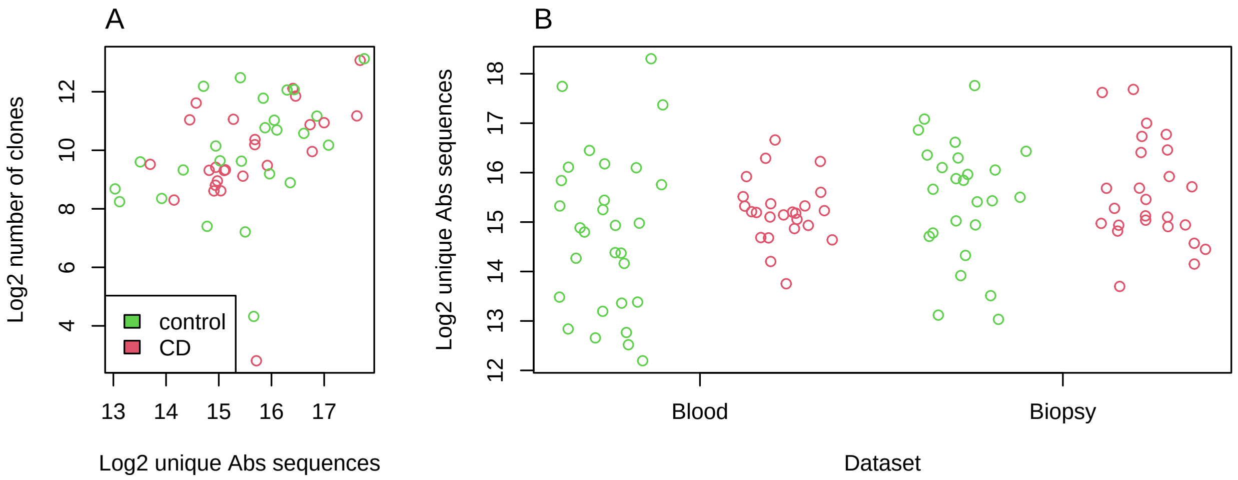

Figure S2: **Technical quality controls of samples used in this study**
A. Plot showing Log2 number of sequences (X axis) of each repertoire in our new cohort, and Log2 number of clones in each repertoire (Y axis). No difference was seen between healthy controls and CD patients. B. Plot showing Log2 number of sequences in each repertoire of our new cohort (right), showing no differences in the number of sequences between CD patients and controls. Similar numbers are also seen in the Bashford cohort (left).

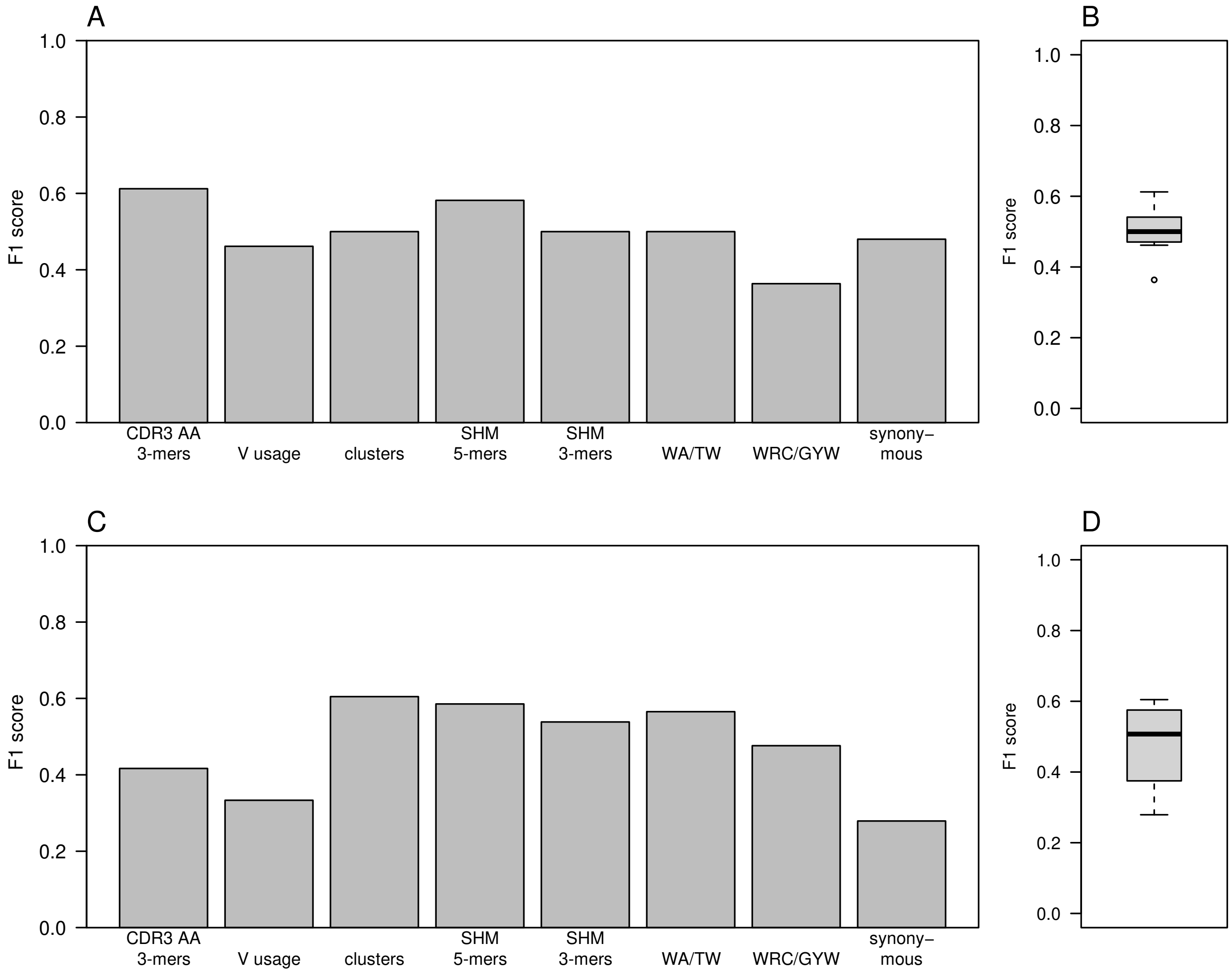

Figure S3: **ML algorithm results on shuffled labels data**
A. The same ML algorithm trained on the true data was trained on shuffled labels data of the DNA cohort. F1 scores of the different runs are shown in barplots. B. Boxplot summarizing the results shown in panel A. C. The same ML algorithm trained on the true data was trained on shuffled labels data of our RNA blood cohort. F1 scores of the different runs are shown in barplots. D. Boxplot summarizing the results shown in panel C.

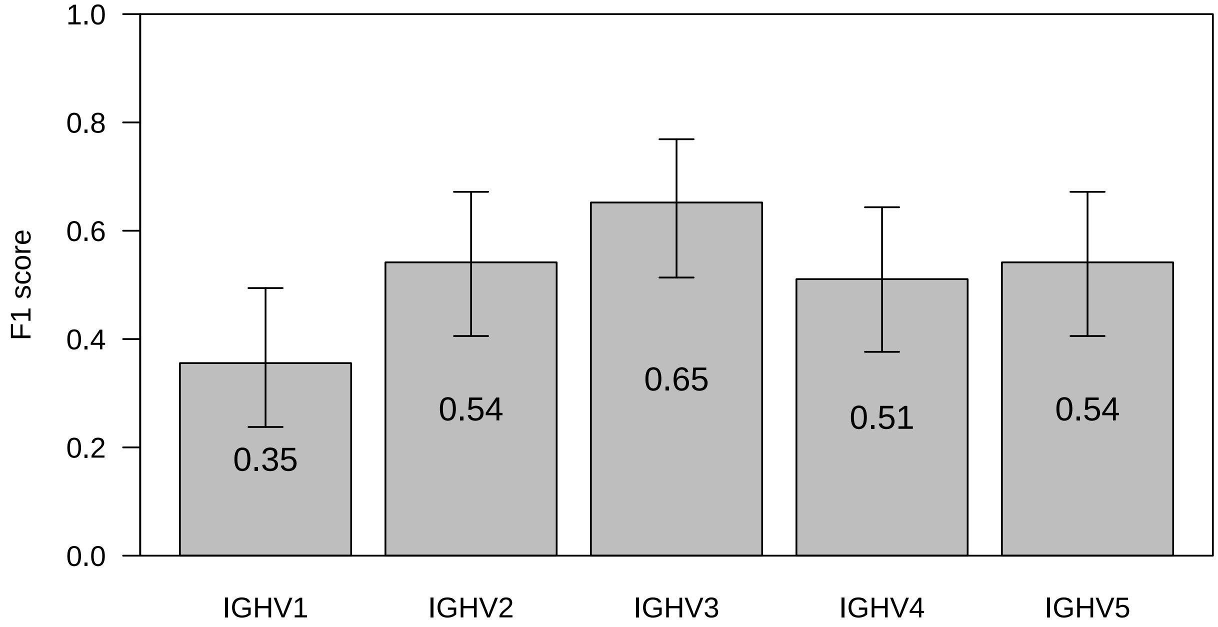

Figure S4: **ML algorithm trained on specific V genes**
The same algorithm used for classification based on SHM patterns in figure 2A was trained on partial data containing only antibodies compromising the indicated V families. Shown are F1 scores estimations based on leave one out. Error bars show the confidence interval of 95%, as calculated using a binomial distribution.

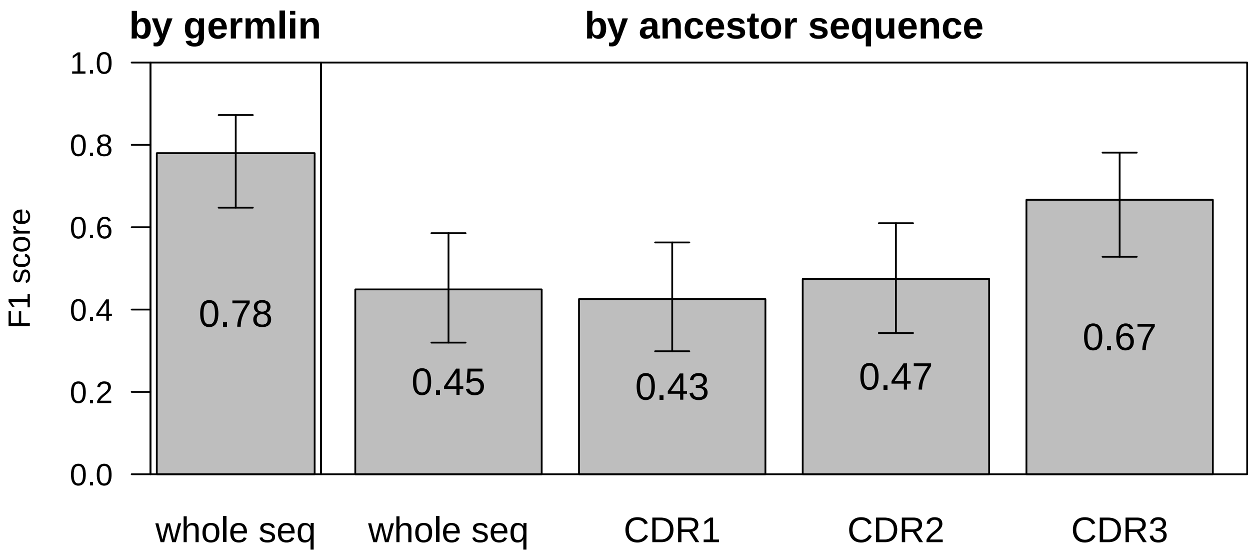

Figure S5: **ML algorithm trained on SHM patterns calculated using linage trees** The same algorithm used for classification based on SHM patterns in figure 2A was trained on a matrix summarizing SHM targeting, which was calculated according to the ancestor sequence in the linage tree of that clone. Matrices were calculated for the whole sequence as well as for the indicated CDR sequences only. For comparison, the left F1 scores were calculated by SHM, which was calculated compared to the germline. Error bars show the confidence interval of 95%, calculated using a binomial distribution.

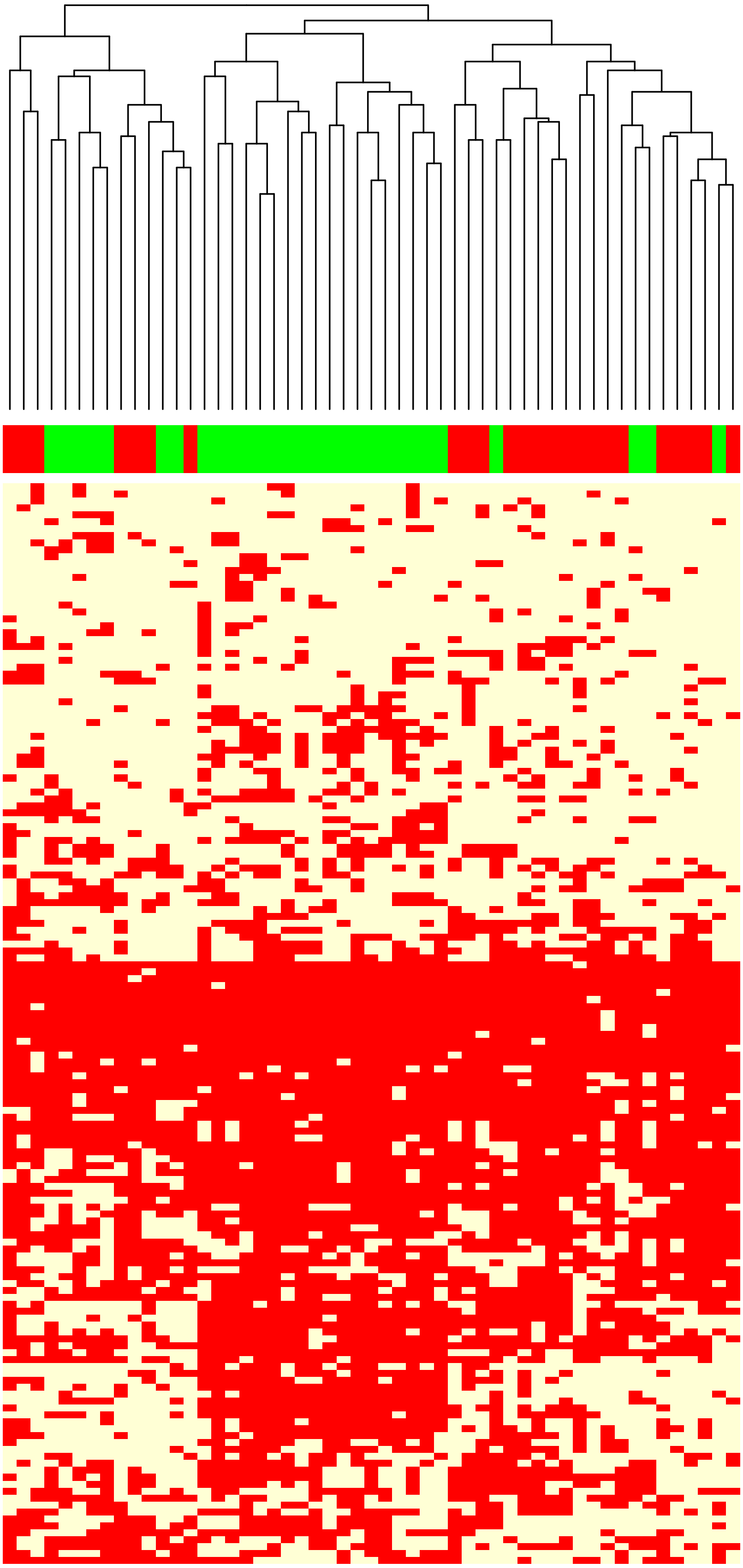

Figure S6: **Allele presence in the RNA-blood cohort**
. Allele presence or absence in each repertoire from the RNA-blood dataset was discovered using the VDJbase pipeline. A heatmap showing alleles present in at least 10 percent of the repertoires, but not in all of them, is shown. Each row represents one allele, each column represents one repertoire. In red are CD patients' repertoires, and in green are controls.

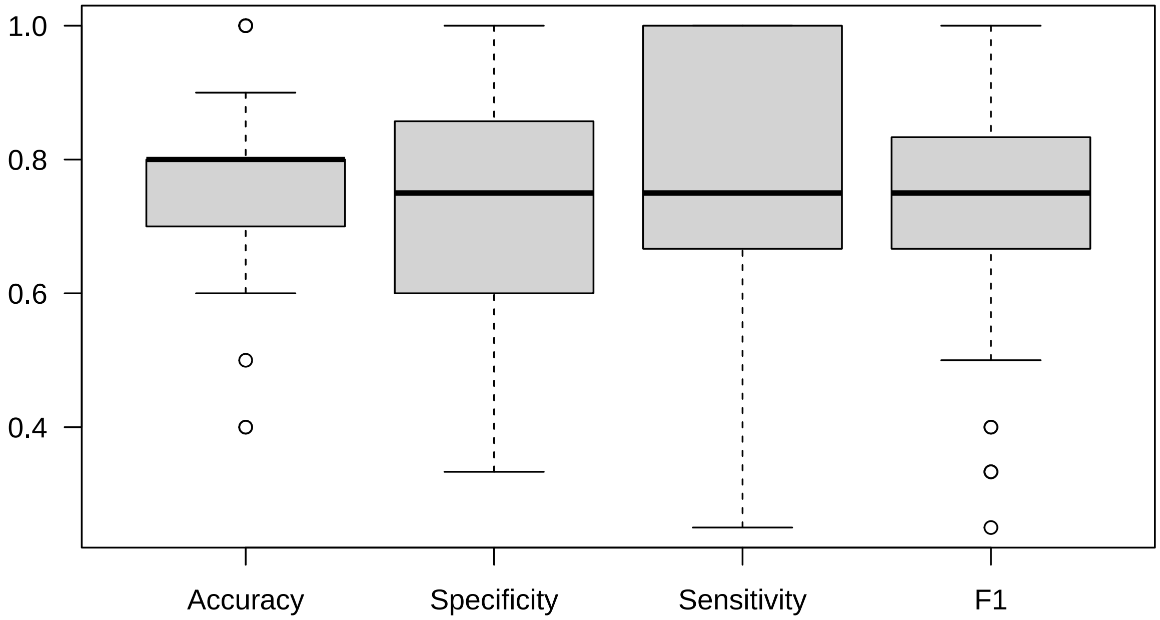

Figure S7: **ML algorithm results for a union of the two cohorts**
Boxplots showing estimations of F1 score, accuracy, specificity, and sensitivity of an ML algorithm trained on an SHM matrix from both datasets. As this combination contains data from 103 repertoires, instead of leave one out, we used 100 random splits on a 93 train repertoire group, and a 10 test repertoire group.

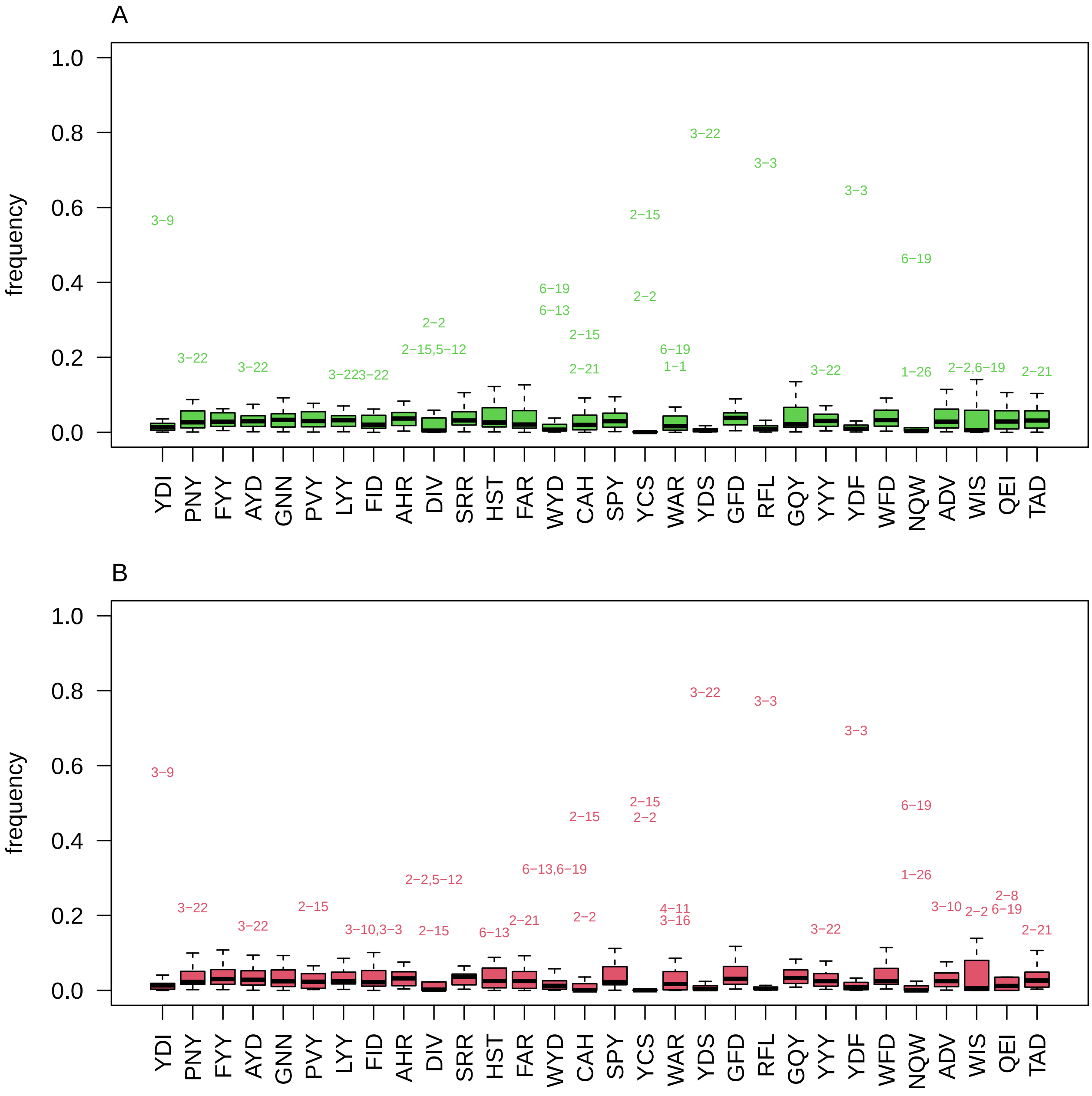

Figure S8: **Connection between CDR3 AA 3-mers and D genes**
Boxplots showing the relative frequency of each CDR3 AA 3-mer in all D genes. Indicated D genes are genes with relative frequency of above 0.15 (close values were merged). In panel A, frequencies were calculated using data from the entire healthy repertoires. In panel B, frequencies were calculated using data from all individuals with CD.
